# Loss of Alzheimer’s disease risk factor *BIN1* from inhibitory neurons induces network hyperexcitability

**DOI:** 10.1002/alz.087069

**Published:** 2025-01-03

**Authors:** Yuliya Voskobiynyk, J. Nicholas Cochran, M. Natalie Davis, Nancy VN Carullo, Jeremy J Day, Erik D Roberson

**Affiliations:** ^1^ University of Alabama at Birmingham, Birmingham, AL USA

## Abstract

**Background:**

Genome‐wide association studies have identified genetic risk factors for AD, including a single nucleotide polymorphism in the *bridging integrator 1 (BIN1)* gene that is present in approximately 40% of the population and has the largest effect size of the common AD genetic risk factors. While the association between *BIN1* and AD has been established, the mechanisms by which BIN1 contributes to AD remain understudied. We previously showed that increasing *BIN1* expression in primary hippocampal neurons increases neuronal excitability (Voskobiynyk & Roth et al., 2020). However, expression of the primary neuronal isoform of BIN1 is reduced in AD patients compared to healthy age‐matched controls. We thus set out to explore the mechanism of BIN1’s contribution to network hyperexcitability *in vivo*.

**Methods:**

We used conditional knockout mice to selectively reduce murine Bin1 from all neurons (*Nestin‐*Cre‐driven), excitatory neurons (*CaMKIIα‐*Cre‐driven), and inhibitory neurons (*Viaat‐*Cre‐driven). We then examined network hyperexcitability through a pentylenetetrazole‐induced seizure susceptibility assay; transcriptomic effects through bulk RNA sequencing, and behavioral effects through a battery of tests.

**Results:**

Pan‐neuronal loss of Bin1 increased seizure susceptibility in a gene‐dose‐dependent manner. Bin1 loss from excitatory neurons decreased seizure susceptibility, while Bin1 loss from inhibitory neurons increased seizure susceptibility. Bin1 loss from inhibitory neurons induced learning deficits and increased mortality of adult mice.

**Conclusion:**

Bin1 loss from inhibitory neurons led to increased seizure susceptibility, similar to the pan‐neuronal loss of Bin1, suggesting that loss of Bin1 from inhibitory neurons drives the effect of global loss of Bin1. Overall, this study contributes to our understanding of how BIN1 regulates network hyperexcitability at a cell‐type‐specific level.

